# Relation-Based Categorization and Category Learning as a Result From Structural Alignment. The *RoleMap* Model

**DOI:** 10.3389/fpsyg.2019.00563

**Published:** 2019-03-20

**Authors:** Georgi Petkov, Yolina Petrova

**Affiliations:** ^1^Department of Cognitive Science and Psychology, New Bulgarian University, Sofia, Bulgaria; ^2^Central and East European Center for Cognitive Science, Sofia, Bulgaria

**Keywords:** categorization, category acquisition, cognitive modeling, analogy-making, context dependence, relation-based categories

## Abstract

Relational categories are structure-based categories, defined not only by their internal properties but also by their extrinsic relations with other categories. For example, predator could not be defined without referring to hunt and prey. Even though they are commonly used, there are few models taking into account any relational information. A category learning and categorization model aiming to fill this gap is presented. Previous research addresses the hypothesis that the acquisition and the use of relational categories are underlined by structural alignment. That is why the proposed *RoleMap* model is based on mechanisms often studied as the analogy-making sub-processes, developed on a suitable for this cognitive architecture. *RoleMap* is conceived in such a way that relation-based category learning and categorization emerge while other tasks are performed. The assumption it steps on is that people constantly make structural alignments between what they experience and what they know. During these alignments various mappings and anticipations emerge. The mappings capture commonalities between the target (the representation of the current situation) and the memory, while the anticipations try to fill the missing information in the target, based on the conceptual system. Because some of the mappings are highly important, they are transformed into a distributed representation of a new concept for further use, which denotes the category learning. When some knowledge is missing in the target, meaning it is uncategorized, that knowledge is transferred from memory in the form of anticipations. The wining anticipation is transformed into a category member, denoting the act of categorization. The model’s behavior emerges from the competition between these two pressures – to categorize and to create new categories. Several groups of simulations demonstrate that the model can deal with relational categories in a context-dependent manner and to account for single-shot learning, challenging most of the existing approaches to category learning. The model also simulates previous empirical data pointing to the thematic categories and to the puzzling inverse base-rate effect. Finally, the model’s strengths and limitations are evaluated.

## Introduction

Imagine showing someone picture of a cat hunting a mouse, pointing to the cat and asking: “What is this?" We can boldly assume that among the first suggestions would be that this is a “cat" and a “predator". The answers will depend on the context construed by why we need to categorize at all, the previous conversation, the respondent’s current focus, etc. Answers, resembling the first one (“cat"), represent the usually called ***feature-based categories***, which have dominated the categorization literature for decades (Rosch et al., 1976; Goldstone, 1994). It is their research that gave birth to the view that categories are defined through their intrinsic properties. Such view would define the concept *cat* as something furry, something that has whiskers and it meows. Yet, usually, those properties are not considered mandatory due to the observation that category membership can be graded (Rosch et al., 1976). That is, a cat that meows would surely be a more typical example of *cat* than a cat that does not meow.

Undoubtedly, the feature-based research approach has advanced our understanding of the way we acquire and use different categories. Despite the differences in the details of how category acquisition and categorization happen, all versions of this approach seem to share the assumption that each categorization decision we make is based on the similarity^1^ between the intrinsic properties of the to-be-categorized entity and those of the categories that we have already acquired (Markman and Stilwell, 2001; Kloos and Sloutsky, 2004).

However, this understanding can hardly satisfy the conceptual richness people hold. If we go back to the imaginary picture of a cat hunting a mouse where the cat is seen as a predator, can we use intrinsic properties to define the category *predator*? Most probably, we cannot, because *predator* is example of a category defined through its extrinsic *relations* with other categories – it is an animal that hunts other animals. It cannot be explained without referring to something outside itself. Recently, this inability has been emphasized more and more gaining a lot of attention for the stand that the categories defined through their properties (the feature-based categories) are psychologically distinct to the categories defined through their relations (the so-called ***relational*** or ***relation-based categories***) (Markman and Stilwell, 2001; Gentner and Kurtz, 2005; Livins et al., 2015; Asmuth and Gentner, 2017). We should emphasize that when we talk about relational categories, the features of the members of those categories have somewhat secondary relevance. If we think of the relational category *predator* for example, although there might be common features among all *predator* members (for example, they are all animals), a *predator* is not defined through them.

Still, one should not remain under the impression that the relational categories are just specific cases which are better researched as exceptions of the pre-dominant in the literature feature-based categories. In fact, Asmuth and Gentner (2017) reported that according to the British National Corpus around half of the most frequently used English nouns have relational meaning. It is the very usage of relational information that allows us to see the analogies between different situations and/or structures of knowledge (Gentner, 1983; Gick and Holyoak, 1983; Doumas et al., 2008). Thus, it is not a coincidence that the importance of the relational categories is becoming more and more recognized (Markman and Stilwell, 2001; Gentner and Kurtz, 2005; Goldwater et al., 2016).

Several types of relational categories could be differentiated. Let us again take the simple situation of a cat hunting a mouse. *Hunts* is a ***relational category*** that relates two arguments in a specific way, namely *hunter* (*predator*) and *hunted* (*prey*). To explain what *hunts* means, one cannot do it only through its intrinsic properties. Inevitably, one will refer to the other categories acting as the relation’s arguments (Gentner and Kurtz, 2005). In turn, the relation’s arguments (*predator* and *prey*) are ***role-governed categories***. By classifying something as a member of role-governed category, it should take a specific role in the relational structure it is being part of Markman and Stilwell (2001). It is the very role in the relational structure that gives the meaning of such categories. Both, the relational and the role-governed categories, are defined as relation-based categories.

However, given the same situation, we can clearly say that the whole situation of a cat hunting a mouse is a ***schema-governed category*** (Gick and Holyoak, 1983; Markman and Stilwell, 2001; Gentner and Kurtz, 2005). The schema-governed categories interconnect relations and objects that those relations take as arguments (Markman and Rein, 2013). Depending on the complexity of the schema, it can contain much larger sets of interconnected relations. A person’s schema for *going to a restaurant*, for example, contains information for many interrelated events – like when and how one can order, eat and pay their bill.

**FIGURE 1:**
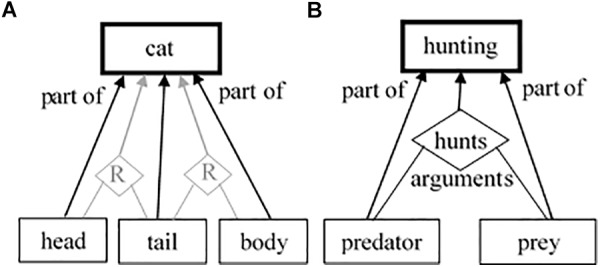
Schematic description of the meaning of a feature-based category *cat* **(A)** and of a relation-based schema for *hunting* **(B)**. The diamond shape *hunts* points to a relational concept. We assume that **(A)** and **(B)** are not two distinct types of categories but are two extremes of a continuum. Even in the *cat* representation **(A)**, we can add some spatial relations between the cat’s parts (All links are bi-directional).

We strongly agree that most schemas are based on relational structures. However, we see these structures as intrinsic, not extrinsic for them and, thus, they could be represented in a similar way to the classical feature-based categories. The main difference is just in the nature of the predominant intrinsic characteristics – whether they are properties or relational structures. The schema-governed category for *hunting*, for example, holds as its intrinsic parts both the relation *hunts* and the role-governed categories *predator* and *prey* (Figure 1). Thus, for us, it seems that what really constitutes the relational categories is their emphasis on their relational structures, rather than on their properties. Probably, there is a whole continuum between the absolute relational categories and the absolute feature-based ones. For example, even an obvious feature-based category like *cat*, could be difficult for describing to an ignorant who does not know what cat is through a pure collection of some parts, without some relations among them. Every cat has a head, a body, etc. Yet, those parts are always interrelated through specific spatial relations. Thus, maybe there are psychologically distinct mechanisms for dealing with relations and with features, but this does not necessarily mean that they reflect any discrepancy between the two types of categories. More probably, the mechanisms for dealing with relations and the ones for dealing with features work together when making a categorization decision for any type of categories.

Finally, there are ***thematic categories*** (see Lin and Murphy, 2001; Estes et al., 2011) whose inclusion in the relational categories is probably the most debatable one. Unlike the role-governed categories, which members always share one and the same role, the thematic category commonality is exhausted to co-occurrence in the structure without the need to take any specific role. Turning back to the example of a cat hunting a mouse, the proponents of the thematic categories argue that the cat and the mouse are members of a such thematic category. There are empirical findings that people’s similarity judgment and grouping preferences increases when the respective items participate as different roles of the same relation, even if they are not part of one and the same situation (Jones and Love, 2007; Goldwater et al., 2016). What puts the existence of the thematic categories into question is that their inclusion depends on no other commonalities between the category members (Gentner and Kurtz, 2005; Goldwater and Markman, 2011). This makes them completely dependent on co-occurrence statistics and, thus, could be better seen as types of groupings not as relational categories (Gentner and Kurtz, 2005). Our view about why we need a conceptual system at all also questions the existence of the thematic categories, which members do not share any similarity at all.

The very basic function of the conceptual system is to provide us with a knowledge which is beyond the perceptual information (Komatsu, 1992). Thus, the categories should have several important characteristics: (1) the members of a given category should share certain similarity. This similarity could vary in complexity – it can be similarity between their properties, but also between their relations, even between their segmentation in time. (2) All concepts share the similar function to keep useful information that may not be available in a certain moment of time. Thus, even though it seems that there are various types of distinct types of categories – feature-based, relational, schema-governed, etc., probably all categories are just points on a continuum between the extremes provided for examples of these types. Most probably, the acquisition and usage of all types of categories share common underlying mechanisms. (3) Context-sensitive categorization is a necessary condition for the conceptual system to fulfill its main function, because this huge number of possible similarities. One cannot consider all of them simultaneously but should choose some of them depending on the context.

That is what we strive to demonstrate – how a context dependent categorization and category learning can be modeled through several basic principles. Importantly, the proposed *RoleMap* model is focused on relational categories, but it is not limited to them. Below, we present the theoretical assumptions the model is based on and several important empirical findings supporting most of those assumptions. We portray the model’s implementation in its current version and then evaluate it against several data sets and well-known psychological effects. The paper ends with a discussion of the eventual implications of the model and its current drawbacks.

## The *RoleMap* Categorization and Category Learning Model

*RoleMap* was developed to advance the understanding of the relational categories specifically, but its abilities surpass that goal. The model assumes that, in general, the categorization is a process of extracting various similarities between exemplars. Its basic idea is to highlight similar parts between objects or episodes, to extract those similarities and eventually to choose one of several possible competing paths – to recognize the new information as something it already knows, to create a new category and to store the new target as its member, or simply to store the new knowledge without categorizing it.

The work in the analogy domain has been convincing that there are at least two types of information which are important for the similarity extraction (Goldstone et al., 1991; Jones and Love, 2007; Livins et al., 2015). The first one considers the place that two items occupy in their corresponding structures and it is called *structural similarity*. The second one considers the properties two items share. We will call it *semantic similarity*. Results from several similarity judgments tasks have showed that when the property matches between two items increases, the judged overall similarity between them also increases. Likewise, independent of the property matches, as the structural match between those items increases, the judged overall similarity between them also increases (Goldstone et al., 1991; Jones and Love, 2007). *RoleMap* assumes that different mechanisms are responsible for extracting the structural and the semantic similarities.

A good candidate to highlight the commonalities between two structured descriptions is the structural alignment processes (Falkenhainer et al., 1989). The first and foremost theoretical stand our model adopts is that the process of structural comparison is the corner stone of wide variety of human cognitive abilities, such as the ability to work with relational information in general (Gentner, 1983), the similarity appreciation (Jones and Love, 2007) and the ability to learn relational categories (Gentner and Kurtz, 2005; Doumas et al., 2008). As a product from the structural alignment different mappings, each capturing a specific commonality, are extracted. The approach was inspired by predictions of the structure mapping theory (Gentner, 1983) which posits that the very alignment highlights the commonalities which sets ground for their abstraction into a relational structure.

The second stand is that the associative organization of the memory serves two functions – it is the basis for the semantic similarity evaluation and, also, it is important for fast and flexible cognition. According to the *RoleMap* model, the semantic similarities could be captured by exploring the associative path length between the items. Associations also reflect the probability of co-occurrence of two items (Anderson, 1996) and prepares the cognitive system for faster information processing. The associative organization of the memory is naturally linked to the context sensitivity, which is a necessary condition for having both flexible and effective cognition (Hofstadter, 2001; Petrov, 2013). For a system to be flexible, it should be able, at least in principle, to explore huge number of opportunities. However, to be effective, it should explore only few of them. The context sensitivity can solve this trade off – in any certain moment the system explores only the relevant for the current context paths, keeping the possibility to explore any path but in appropriate context.

These assumptions (sensitivity toward the relational structures and toward the context) are already incorporated in the cognitive architecture *DUAL* (Kokinov, 1988; Kokinov, 1994; Petrov, 2013). Thus, we chose to implement *RoleMap* in *DUAL* by keeping most of the architecture’s mechanisms and adding only few additional.

We assume that the perceptual system has the ability to recognize some relations. Thus, when we encode a new knowledge base, we start from the highest order relations, continue with the lower ones and finish with some primitive elements. In a clear-cut sense, when the model is presented with a new situation on the input, it has a single task – to find its best long-term correspondence by considering both semantic and structural similarity. The model’s categorization decision occurs during this very search. Importantly, all mechanisms implemented in *RoleMap* overlap in time and, thus, constantly influence each other. The model’s behavior does not result from a central executor but emerges from a huge number of local interactions between many agents. However, observing its global behavior from outside, it may look like the model calculates similarities between the target and the relevant for the current context memories (considering both semantic and structural information), and takes decisions based on those similarities.

We begin the more detailed portrayal of the model with a brief description of its knowledge representation and what it needs to start its similarity search. Then, we explain what influences the model’s work and the implementation form of those pressures. The section ends with an outline of how those paths compete for the model’s final categorization decision.

### *RoleMap*’s Knowledge Representation^2^

Roughly speaking, there are several specifics concerning the *RoleMap*’s knowledge.

**FIGURE 2:**
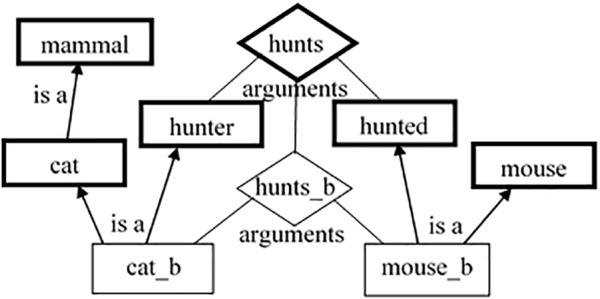
Schematic description of part of the *RoleMap*’s knowledge. The diamond shapes point to relations; the bold rectangles – to concepts; the thin rectangles – to instances (All links are bi-directional).

**FIGURE 3:**
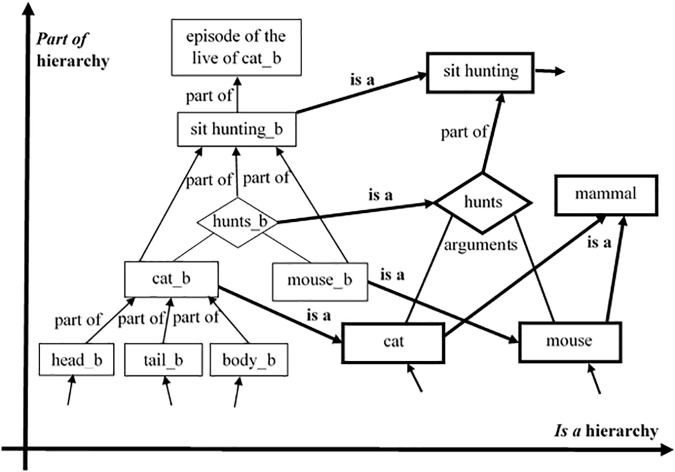
Part of the *RoleMa*p’s knowledge representation, representing the two-dimensional structure through the *is a* and the *part of* hierarchies. The bold items point to concepts (All links are bi-directional).

**FIGURE 4:**
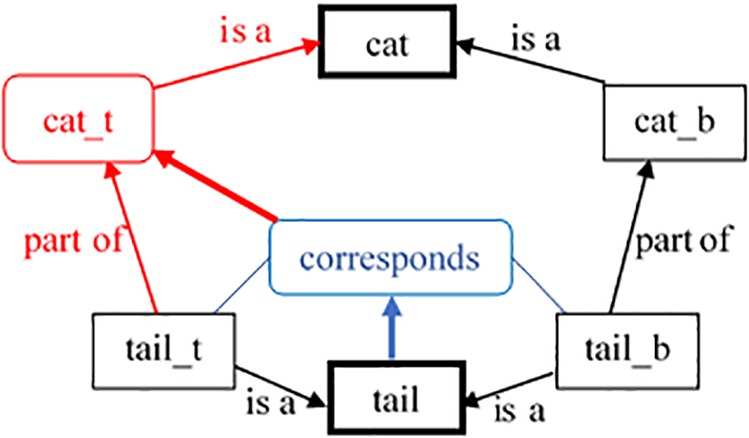
Illustration of mapping agents (in blue) and anticipation agents (in red) in the *RoleMap* model. The correspondence (mapping agent in blue) agent has the concept *tail* (the lower bold rectangle) as justification (pointed by the bold blue arrow). The mapping itself is a justification (bold red arrow) for the anticipation agent *cat_t* (marked in red).

(1) When the model is instantiated, its knowledge consists of hand-coded *semantic* and *episodic* agents, resembling the *type-token* differentiation (see Kahneman and Treisman, 1984). Each agent carries knowledge about a single *distinct entity* (Hofstadter, 2001).
(2) The agents are connected to each other with various types of links, thus, composing structural descriptions. There are links expressing semantic and structural connectedness. The semantic connections are realized through links such as *is_a*, *instance_of, part_of, has_part*, etc. They make sure that episodic agents (for example, *cat_b*) point to their concept (*cat*), which point to their own superclasses (*mammal*) and vice versa. The structural connectedness is expressed mainly through the links between the relations and their arguments (or roles). Such representation is isomorphic to the predicate representation, for example, in the *SEQL* model of categorization (Kuehne et al., 2000) but allows for adding weights to these links (see below) and hence, for a context sensitiveness, which *SEQL* lacks. Taking again the situation of a cat hunting a mouse, the episodic relation *hunts_b* will have bidirectional links to its episodic arguments – *cat_b* and *mouse_b*. Those links capture the knowledge that all relations go hand in hand with their arguments and can hardly have any meaning without each other (Markman and Stilwell, 2001; Goldwater et al., 2011). In addition, there are *is_a* links from *cat_b* to the concept *hunter* and from *mouse_b* to *hunted*, respectively. The role-governed categories *hunter* and *hunted* are themselves arguments of the relational concept *hunts* (see Figure 2).

(3) The third peculiarity stands that those descriptions and all other *RoleMap* agents form two hierarchies – the abstractness hierarchy and the part-of hierarchy. Figure 3 demonstrates how the situation from the beginning of the paper – a cat hunting a mouse – could be structurally represented together with its most highly associated knowledge. Having concepts on different levels of abstractness allows the cognitive system to keep the balance between generality and specificity (Tenenbaum et al., 2011). More general concepts would mean more cognitive economy. But if our concepts are too general, we would be able to make fewer inferences. Vice versa, if our categories are too specific, we will be able to make more specific inferences, but this would be too cognitively expensive (Komatsu, 1992; Tenenbaum et al., 2011) and will not allow us to make abstract cross-domain analogies. From the other side, the part-of hierarchy allows *RoleMap* to combine the advantages of distributed and localist representations, similarly to what the *DORA* model (Doumas et al., 2008) does. It represents every object, situation, etc., distributed but at the same time it keeps a binding-node at the higher level of the part-of hierarchy.

(4) Each agent is treated either as a target or as a base one. The target agents reflect the current situation – the environment and the goals^3^ of the system. The base agents account for the past and consist of all other declarative and episodic memories.
(5) In addition to the permanent (*semantic* and *episodic*) agents, *RoleMap* itself creates temporary agents. The temporary agents are *mappings* and *anticipations* found and made by the model. Each mapping agent represents a single semantic or structural commonality between two episodic agents. Based on the created mappings, the model makes hypotheses in the form of *anticipation agents* concerning some missing information in the structure of one side of the mappings, including the category membership of the new episode (Figure 4).

(6) Finally, all permanent agents, hand-coded by the modeler, and all temporary agents, created by the model itself, are hybrid in nature. What makes the *RoleMap* agents hybrid is that in addition to their symbolic part, they all have a connectionist part, which allows them to impact the other agents. Every agent has *activation level*, representing its relevance to the current context, which changes dynamically reflecting the changes in the environment or the goals of the system. The activation spreads in the network of agents through the links as in a classical connectionist network. With respect to the connectionist aspect, the type of the links doesn’t matter, only their weights do.
(7) The associative organization of the memory is usually assumed to reflect the probabilities of co-occurrence in the nature (see Hinton and Anderson, 2014) which change slowly in time. All links in *RoleMap* also stand for *associations* between agents. Mechanisms of slow gradual change of the weights (for example, kind of Hebbian learning) will strengthen *RoleMap* and will allow it to account for a broader range of behavioral data. However, such mechanisms are still not implemented in the model and all links from one and the same type have equal weights, predefined as parameters. As mentioned before, *RoleMap* assumes that its mechanisms do not work in isolation but influence each other highly. Thus, by excluding the slow associative learning we can better understand the role of the other mechanisms.

To give some sense of that schematic description, next we will describe the mechanisms of *RoleMap*, together with the cognitive pressures, which they reflect, as well examples how the model’s behavior emerges from the work of the agents themselves.

### *RoleMap*’s Mechanisms in More Details

The way we assume the main work of the cognitive system in general is that the new information constantly tries to fit to the old one both because of semantic or structural similarities. Usually in any time there is a lot of unavailable information and the memory is used to fill the holes with expectations about it. Categorization occurs while these general cognitive processes work; it is not a separate process done in a special learning mode. The mechanisms described below reflect this view.

The main mechanisms of *RoleMap* are:

(1) *spreading of activation and associative-driven retrieval*. As it was mentioned, all agents are hybrid in nature, combining symbolic and connectionist part. The pattern of the activation level of all agents represents the current context and it changes dynamically like in a classical neural network, representing the changes in the context.

Immediately after the model receives a new situation, the agents representing that situation turn into constant activation source and are marked as target agents (see *RoleMap*’s Knowledge Representation point (4) above). The activation flow travels through their links to other agents, which in turn activate agents connected to them, etc.

Each agent has its own activation level which is recalculated every cycle (see Supplementary Material for the full verbal description of the model and the parameters values). During some of the simulations, however, to simulate random variations of the knowledge base, we added a random noise to the weight of the links.

When agents receive considerable activation pressure (i.e., their activation level exceeds a predefined threshold), it means that they are context relevant, which is why they become part of the model’s active memory. Because the input agents hold the first activation, the first retrieved agents are usually the ones that are closer to them. The retrieval is essential for all the other pressures, because they can impose their influences only to agents from the active memory. That is due to the fact that the best correspondence to the novel situation is searched only among the active agents.

(2) and (3) Two complementary pressures strive to find *semantically similar* and *structurally similar* correspondences between all episodic agents representing the new situation and all episodic agents retrieved from the memory (respectively, *target* and *base* agents). The first one is responsible for finding agents that share the same category. Each concept that enters the active memory looks for active members – both its direct members or members on lower level of abstractness. Thus, it collects pairs of target-base instance correspondences. Each instance that enters the active memory undergoes a similar process – it seeks a path through the active part of the hierarchy for a corresponding instance. Meanwhile, the second pressure, known from the analogy-making domain, is responsible for finding agents participating in corresponding structures (Gentner, 1983; Holyoak and Thagard, 1989). The structural consistency [following the well-known *systematicity* pressure (Gentner, 1983)] is achieved through making sure that the arguments (roles) of all corresponding relations also match independently of their semantics – i.e., when two relations are in correspondence, the model creates correspondences for their respective arguments too.

**FIGURE 5:**
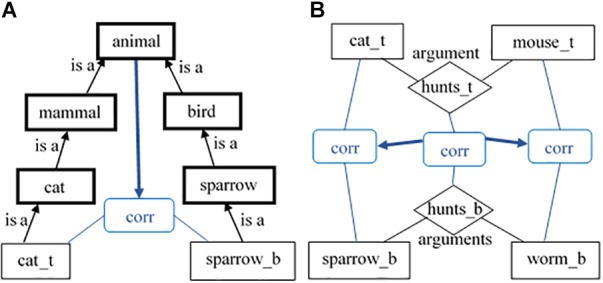
Mappings (in blue) created by **(A)** semantic and **(B)** structural pressures together with their respective justification links.

When a correspondence is found, the model creates a mapping (correspondence, abstraction) agent that captures and stands for that commonality. The mappings represent twofold knowledge – from one side, they reflect analogical correspondence between items and from the other side, they are proto-abstractions, which means that by representing commonalities between items, they stand as a justification to abstract the corresponding episodes (Figure 5).

From a connectionist point of view, the mapping agents receive activation (relevance) by the two agents that formed it and by the agent which was the reason for their correspondence – if the similarity is semantic, that would be the semantic agent they share as concept; if the similarity is structural, that would be the mapping agent between the two relations they are arguments to. When two episodic agents share both semantic and structural similarity, the mapping referring to their correspondence receives activation from more agents, representing both semantic and structural activation pressure in the form of supporting links. In addition, again following the *systematicity* pressure (Gentner, 1983), supporting links among the mappings from one and the same structure (i.e., a set of interconnected with relations agents) are created. That means that the similarity between bigger structures will receive more supporting links, meaning that its influence will be more powerful.

The newly created mapping agent in turn influences the activation level of the corresponding agents by making them even more relevant. In addition, they also influence the agent being the reason for their correspondence – the shared semantic agent (for semantic correspondences) and the mapping agent reflecting the correspondence between the two relations (for structural correspondences) (see Figure 5). This is how the mappings influence the retrieval process. Their activation pushes for further retrieval of information connected to the corresponding agents.

In addition to all supporting links, there are also inhibiting temporary links. They emerge during the work of the model and embody the famous *one-to-one pressure* (Gentner, 1983), also known from the analogy-making domain. This is the pressure each agent to be mapped to at most one other agent from the model’s active memory. That is why all mappings that share the same input agent contradict and thus inhibit each other.

(4) *Categorize similar things as belonging to the same category* *through* *anticipation formation*. Importantly, the created mapping agent checks whether it may contribute for filling any missing information in the target. It goes through the links of the base agent and checks whether its neighbors have respective correspondences in the target. If not, it creates anticipation agents and supports it (or it just supports if such anticipation is already created). For *RoleMap*, the anticipations of interest are those that categorize the target elements. If a part of the distributed representation of something is mapped to a part of already categorized item (object, situation, etc.), an anticipation for the category of this item is created (see Figure 4).

The anticipations are proto-instances. They represent the knowledge that some elements could be combined as belonging to one and the same thing (object, situation, etc.). In this way, the anticipations are proto-binding nodes for uncategorized distributed representation of something.

If there are more mappings that justify one and the same anticipation, they all support it. If there are competing anticipations for one and the same item, they inhibit each other, expanding the one-to-one principle (Gentner, 1983) to the anticipations. In this way, the anticipations in turn influence the spreading of activation and retrieval, as the mappings do.

As it was mentioned, all agents work locally without any central executor. Thus, each part of a certain object may create its own anticipation (for example, if there are a *head* and a *body* presented on the input, the mappings for the *head* may create an anticipation that this *head* is part of a *cat*; the mappings for the *body* – another anticipation for a *cat*). The different anticipations for one and the same thing should be combined. However, how to distinguish when the two elements are parts of one and the same object from when they are parts of two different objects? (Considering the example above – how to know whether there is one cat on the input and we see its head and body or there are two cats and we see the head of the first one and the body of the second one, because they are partially occluded?) The solution of this problem, following the empirical findings of Corral and Jones (2014), in our implementation is to combine all anticipations that are about parts, interconnected with relations. In the example above, it is assumed that the *body* and the *head* of a certain *cat* should be connected through a specific spatial *relation*. That way, all three agents – the *head*, the *body*, and the *relation* among them, are interconnected, thus would combine their anticipations. However, this is a much deeper and general problem, concerned with how people separate object from background and how they segment the time into events and situations. The *RoleMap* model doesn’t resolve this problem, it assumes that the perceptual system somehow does this. Probably, there are a lot of indications for separating figure from background. *RoleMap* model simulates only one of them – it assumes that if the elements are interconnected with relations, most probably they are parts of one and the same object.

(5) *Combining various pressures into a constraint satisfaction network*. From the connectionist point of view, all those pressures incrementally construe a constraint satisfaction network of supportive and inhibitory links between all mappings and anticipation agents (Holyoak and Thagard, 1989), which is itself incorporated into the main knowledge network. Due to the different pressures, the active memory is dynamic – agents are going in and out from the active memory and hence, new mappings and anticipations emerge in time. What the network tries to do is simply satisfy as many of its acting pressures as possible. The important difference here is that because they are pressures, at least in theory, each pressure can be violated if the other pressures push to a contradicting behavior. None of the pressures is a deterministic rule, rather, the interplay between all of them dynamically shapes the course of the model’s behavior (Hofstadter, 1984). The tenet that both categorization and category learning process rely on all the listed pressures is central for understanding the model’s idea.

In summary, the constraint satisfaction network combines the links between the permanent agents from the main network (*is_a*, *part_of*, *argument_of*, etc.) with the following links to and from the temporary agent (Table 1):

**Table 1:** The set of situations used for testing *RoleMap*’s stability.

Set	Base situation 1	Base situation 2	Target situation
First set	*Grandmother feeding a chicken.*	*Grandmother feeding a pig.*	*Grandmother feeding a cow.*
Second set	*Grandmother feeding a chicken.*	*Neighbor milking a cow.*	*Grandmother feeding a horse.*
Third set	*Grandmother feeding a chicken.*	*Neighbor milking a cow.*	*Neighbor feeding a horse.*
Fourth set	*Grandmother feeding a chicken.*	*Neighbor milking a cow.*	*Girl brushing a horse.*

(6) *The act of categorization or creation of categories.* The constraint satisfaction network guides two contradictory tendencies. From one side, the model tends to use its old knowledge and to categorize the current situation into something it already knows. From the other side, it tends to create new concepts, which reflects the learning of something new.

These two tendencies are implemented in *RoleMap* by a competition to turn the anticipations into binding-nodes and to turn the mappings into concepts. The competition is resolved by the activation levels of the respective anticipations and mappings, guided by the constraint satisfaction network. If either a mapping or an anticipation satisfies the respective conditions to be transformed, then the model makes a categorization decision. If a mapping reaches the respective threshold before the respective anticipation does, this will lead to a new category creation. Vice versa, if an anticipation wins, this will result in a categorization into already known category.

When the activation level of an anticipation agent exceeds a certain threshold (fixed to 10 in the simulations), it is transformed into a permanent instance agent. This new instance agent stands for a binding node for the elements on the lower level of the part-of hierarchy, which mappings justify it. After this transformation, the model does some additional work to achieve consistency. The elements from its distributed representation adjust their *part_of* and *is_a* links consistently and their mappings are stopped from their tendency to become permanent concepts (Figure 4).

**FIGURE 6:**
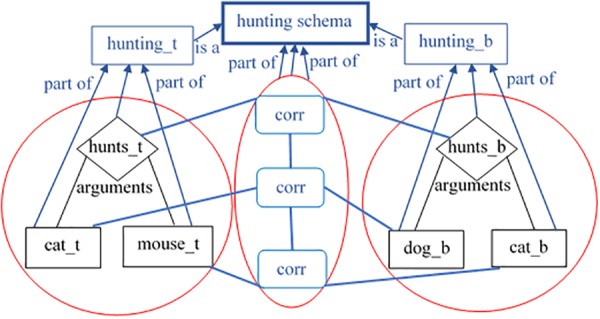
Creation of a new schema concept called *hunting schema* with two instances (*hunting_t* and *hunting_b*). The schema creation happens through transformation of mapping agents (the shapes in blue called *corr*) into concepts. The mappings themselves are transformed into a distributed representation of the new schema concept. The mapped elements from the target and the base form two instance agents, which are the first instances of the new schema concept. The red circle on the left refers to the initially encoded target situation. The red circle on the right refers to the initially encoded base situation. The middle red circle covers all mappings generated by RoleMap.

When the activation level of a mapping agent exceeds its respective threshold (again, fixed to 10), it is transformed into a permanent concept in the respective level of the class-hierarchy. The supplementary work after this transformation includes cleaning up of the competing mappings and anticipations. In addition to this, it supports the transformation into permanent concepts of the other mappings capturing commonalities between the other agents, which are interconnected with relations with the winner (Figure 6). All these newly created concepts form a coalition, which is the distributed representation of a new entity, which keeps the relational structure. The model creates one additional agent, representing this entity – capturing the whole schema. The schema might be thought of as kind of binding node for the lower level concepts of the part-of hierarchy. Thus, when such an concept is created (representing a schema-governed category), it emerges both with its distributed and localist representation. The target and its corresponding base become their first exemplars; their parts become the first exemplars of the respective concepts from the lower level of the part-of hierarchy. In this way, even though that *RoleMap* seems similar to the *DORA* model (Doumas et al., 2008) in the sense that distributed and localist representations are combined, it differs largely from it in the assumption that relational roles and the relations themselves emerge together, at one and the same time. Empirical support for this assumption is provided by Goldwater et al. (2011).

The next section aims to demonstrate how the described pressures work in practice. We start with few simple simulations, testing whether the implemented mechanisms work as they are supposed to. We describe a simulation of the way *RoleMap* learns new concepts and how it uses those concepts for further categorization; then we explore the role of the associative organization of the memory and demonstrate the model’s context sensitivity. Finally, we test the structural representation of the knowledge and more precisely, how it allows the structural consistency pressure to prevail over the semantic similarity one when trying to find the most appropriate correspondence to the target agent.

Then, we propose a simulation of empirical data which were interpreted as role-governed and thematic grouping preference (Goldwater et al., 2016). On one side, the simulation demonstrates extraction of both role-governed and thematic information from the same set of events. On the other, it shows how the obtained psychological results arguing for thematic category existence, can be explained as emerging from associative closeness, without the explicit postulation of thematic categories. The section ends with a simulation of the classical inverse base rate effect (Gluck and Bower, 1988), which demonstrates the work of the constraint satisfaction network in more detail.

## Verification Simulations – the Interplay Between the Pressure to Categorize and the Pressure to Learn New Categories^4^

### Proof of Concept Simulations

#### Learning Role-Governed Categories and Schema-Governed Concepts

To be easily traceable how the pressures come into play, so the model can learn and categorize, we simulated a simple set up. We provided the model with a single base episode of “*A neighbor milking a cow*.”^5^ This means, we created three episodic agents for *RoleMap* – the relation *milks*, and its arguments *neighbor* and *cow*, and interconnected them with appropriate links. As prior semantic knowledge, we encoded concepts for *neighbor*, *grandmother*, *cow*, *pig*, *chicken*, *animal*, etc., and relations such as *feeds* and *milks*. All agents, including the episodic ones, were interconnected with their respective *is_a* and *arguments* links.

The moment we introduced the model’s input with a target situation of “*A grandmother feeding a chicken*,” due to the associatively driven retrieval, the base episode of “*A neighbor milking a cow*” became active. This allowed the *semantic similarity pressure* to create a mapping between the *grandmother* and the *neighbor* – because they share the concept *person* upper in the hierarchy. Similar semantic commonalities were established for the relations *feeds* and *milks* (as types of *care*), which in turn let the structural pressure to make sure that their corresponding roles are mapped as well. Thus, the mapping between *grandmother* and *neighbor* (accordingly, *chicken* and *cow*) became justified by two reasons – their semantic similarity and their structural similarity as performing similar roles in the structures they were part of.

The knowledge of the described simulation consisted of a single base episode. Because all mappings supported each other (being in the same structure) and had no competitors they all reached the transformation threshold. For each mapping exceeding that threshold, *RoleMap* underlined the commonality between the mapped elements and asked the user for a name of the concept which was to be created, so it received the names *domestic animal* (for the mapping between the *chicken* and the *cow*), *animal care* (for *feeds* and *milks*) and *animal caregiver* (for the mapping between the *grandmother* and the *neighbor*) ^6^.

The new concepts *domestic animal* and *animal caregiver* are examples of role-governed categories. In addition, the model created a new “schema-governed” concept representing the common parts of the two situations. We called it – *domestic animal care*. The two mapped episodes became the first two episodic instances of that schema. Accordingly, the three newly created concepts – animal care, animal caregiver and domestic animal – became parts of the “schema” (with bi-directional links to and from it). Note, the binding node for the schema emerges together with the role-governed categories, which serve as a distributed representation of the schema, contrary to the *DORA* model (Doumas et al., 2008), which learns relations only after initially the roles are abstracted on the basis of feature similarity alone. In this way, *RoleMap* coincides with the empirical data of Goldwater et al. (2011). Even though the concepts above were created from mappings, once they were transformed into concepts, they could have and did affect the subsequent behavior of the model, just as the rest of its (hand-coded) semantic knowledge.

#### Categorization Through Newly Learned Categories

*RoleMap* continued its work and demonstrated its ability to use what it has learned by categorizing a new target situation. The previous target episode of “*A grandmother feeding a chicken*” and the new concepts were already incorporated in the model’s long-term knowledge. In that moment we gave to the input a new target episode – “*A girl brushing a horse.*” Analogously to what was described above, the agents, representing the new target started activating other agents and some mappings appeared. Contrary to the previous simulation, there were competing mappings and they inhibited each other (because of the 1:1 correspondence pressure) which decreased their activation level. The mappings between the *girl* on the input and the base *grandmother* and between the *girl* and the base *neighbor* both created an anticipation that the whole target episode is instance of the *domestic animal care* schema (which was just learned). All mappings underlying commonalities between the new situation and the two retrieved situations supported that anticipation. (During this simulation, there were no competing anticipations. If there were, they would have inhibited each other as well. In the same way, as with the mappings’ competition, the interplay between the supporting and inhibiting each other anticipations would have resulted in a single winning anticipation.) When the anticipation became active enough, it was transformed into an instance, meaning that the model made a categorization (In a certain context it may happen that some of the mappings win before the anticipation. In such case, a new category will be made). Subsequently, the model incorporated the new target episode by correctly adjusting it as an instance of the *domestic animal care* schema-governed concept. The corresponding bi-directional links were set up accordingly for all target parts as well. In that case, the *girl* on the input was categorized as an *animal caregiver*; the relation *brushes* became instance of *animal care* and the *horse* was categorized as a *domestic animal*.

In this way, while the first simulation demonstrated the ability of *RoleMap* to learn new concepts, the second one shows how these new concepts could be used further for categorization.

#### Testing the Model’s Stability

To explore the stability of the model, we created several sets of situations (each set contained three situations about people caring for different animals). The sets were run sequentially, presenting the three situations one by one. The model always stored the first situation; created new concepts during the second; and finally, the third situation was either categorized as member of the already created schema concept, or created a new schema, combining the third situation with one of the two situations, presented before that. We explored exactly this decision of the model – whether it will categorize the third situation, or it will form a new category. The categorization decision depended on the competition between the anticipations (pressuring the model to categorize) and the mappings (pressuring the model to create new categories) – which will pass its threshold first. The sets of situations differed according to the similarities between the situations (Table 1).

In the first set there were three highly similar situations. In the second set the first two situations were relatively different, while the third situation was similar to the first one. This simulated initial creation of a sparse concept followed by a situation more similar to one of the two categorized situations. In this case we expected higher tendency to create new sub-category, combining the two similar exemplars, diverging them from the different base. In the third set, we changed the third situation only (the target), making it a combination of the other two. Thus, we expected the tendency for categorization to increase. Finally, in the fourth set we again kept the same two bases and introduced a target situation, very different from both bases. The simplicity of the bases was deliberate. We wanted to explore the influence of a single similarity or dissimilarity, namely the length of the path between them through the class hierarchy. However, any other similarities or dissimilarities between the entities influence the work of the model in the same way.

To obtain statistical results, we run each of the sets 100 times, by adding random noise in the strength of all links of each situation [noise from N(0, 0.25)], as well as in the initial activation of all agents [noise from N(0, 0.05)]. We counted the percentage of times the model categorized the third situation instead of creating new concepts between it and some of the base situations (i.e., the anticipation won before the mappings). For the first set (the control one) the parameters were fitted to produce 75% of categorization, so the tendencies from the other sets could be explored.

The results were as follows: the model categorized the third situation in 11% for the second set; in 30% for the third set; and in 52% of the cases for the last one (Note that the exact values do not have much sense, only the tendency does). Thus, the results confirmed our expectations – the number of categorizations increased when all situations were different. The categorization decreased (in exchange for the formation of new categories), when the target situation was similar to only one of the base situations.

#### The Roles of Context Sensitivity and Structural Pressure

One of the fundamental corner stones of *RoleMap* is its context dependence (in contrast to *SEQL* Kuehne et al., 2000, for example).

In a very simple simulation, we created a knowledge base consisting of two situations only. The first one consisted of a schematic representation of a *cat*, which *eats meat*. It was just a coalition of agents representing the parts of the *cat* (*head*, *tail*, *pawns*, etc.), interconnected with relations and three more agents, pointing to the property that it *eats* *meat*. This coalition had no binding node, i.e., the object was not categorized. The second situation represented a *penguin* (with *wings*, *feathers*, *body*, etc.), again uncategorized.

We gave to the model’s target a schematic description of a *falcon* – represented through a coalition of agents that share some features with the *penguin* (as *wings*, *feathers*, and *body*) and also the information that it *eats meat*. Two sets of inter-supporting each other mappings emerged – between the common parts of the *falcon* and the *penguin*, and between the part of the *falcon* description and the *cat*, pointing to the fact that they both *eat meat*. Then just by adding additional activation on some concepts, we variated the current context – either additionally activating the concept *eats*, or *body*, simulating different attentional focuses. Not surprisingly, in the first case the mappings between the target and the *cat* prevailed quicker and a new concept, generalizing the *cat* and the *falcon* emerged. The distributed representation of this new concept consisted of the winning mappings, which were about the relation *eats* and its arguments. When the system “asked” for a name of the new concept, we called it “*carnivore*.” In the second case, when the activation of the body was pre-activated, the other part of the mappings reached the decision threshold first. The distributed representation of this new concept consisted of concepts such as *feathers*, *wings*, etc., and we gave it the name “*bird*.”

The context sensitivity of the model emerges naturally from the spreading of activation mechanism. All competing mappings, anticipations, etc., receive activation from their elements the more relevant something is, the higher support it gives to the mappings and anticipations it is consistent with. Any emerging competition is resolved by a constraint satisfaction network, which is gradually built by various pressures. Among the most important of them are the pressures for structural consistence and for semantic similarity. In the next simulation we highlighted the different outcomes that they produce.

#### Cross-Mapping

To compare the strength of the structural and the semantic pressures, we can have a setting like the following one. If we say that “*Jupiter revolves around the Sun, because of the Sun’s greater mass*” and “*Io revolves around Jupiter, because of Jupiter’s greater mass*”, then *Jupiter* is in a situation of cross-mapping (Markman and Gentner, 1993). Considering the property similarity, then *Jupiter* from the first sentence is very similar to *Jupiter* from the second sentence – actually, they are the same, thus sharing all the superficial features and intrinsic properties they could. But, if we consider the structural similarity, then *Jupiter* from the second sentence is more like *Sun* from the first sentence, because they both revolve around something. Such examples show that different similarity measurements lead to different outcomes. Experiments using that kind of settings in which participants should match similar objects from two scenes show that at least 60% of the subjects prefer structural instead of property similarity (Markman and Gentner, 1993).

To simulate such cross-mapping situation, we designed a very simple structural description of the fact that *Jupiter revolves around the Sun* (consisting of the relation *revolves* with two arguments, pointing to its respective concepts – *Jupiter* and *Sun*) and added it to the long-term memory of the model. Then we gave to the model as target a representation of the information that *Io revolves around Jupiter*. Two mappings for the target *Jupiter* emerged – one with the base *Jupiter*, supported only by the concept *Jupiter*, and one with the base *Sun*, supported by two agents – the mapping between the two relations (*revolves* from the base and from the target, respectively), and by the role concept *“something one revolves around*.*”* Thus, everything else being equal, the second mapping, capturing the structural commonality, prevailed against the one, capturing the semantic similarity. What that means is that the activation of the mapping between *Jupiter* and *Sun* reached the transformation threshold earlier than the mapping between the two examples of *Jupiter*. However, this behavior of the model resulted from a pressure, not from a deterministic rule, meaning that the structural preference could be overridden by the semantic one. To demonstrate that, we varied randomly the weight of all links, adding to their predefined weight a noise from N(0, 1.00) and to the initial activation of all agents [N(0, 0.15)], and ran the simulation 100 times. The structural pressure won against the semantic one in 74% of the cases, meaning that in those cases the target *Jupiter* was found to better correspond to the base *Sun*. When we repeated the simulation another 100 runes but adding initial additional activation to the concept *Planet* (superclass of the concept *Jupiter*), simulating attention focusing to a specific semantic aspect, the structural pressure prevailed only in 15%s of the cases.

#### Learning a Role-Governed Category and a Schema-Governed Category Through Episodes With Higher-Order Relations

*RoleMap* can also deal with more complex structures containing higher-order relations. To demonstrate that ability, we gave the episode of “*A grandmother feeding a chicken which causes the chicken to give eggs*.” on the input. The episode “*A neighbor feeding a cow which causes the cow to give milk*.” was encoded as base, together with concepts such as *grandmother*, *chicken*, *cow*, *milk*, *eggs*, and the relational concepts *feeds*, *causes*, as well as *gives*. Importantly, the relational base instance *feeds* and the relational base instance *gives* were both encoded as arguments of the relational base instance *causes*. The target episode was represented in an analogous way. As in all previous simulations, when the target agents appeared on the input, the spreading activation mechanism caused the relevant base agents to become active one by one. Thus, various mappings between the instances started to be created – total of 6 mappings emerged. They all survived long and active enough to exceed the upper activation threshold and were transformed into concepts (*animal feeding, animal feeder, domestic animal, animal product*). In addition, a new relational concept was created (*gives food* with *domestic animal* and *animal product* as arguments). This concept was itself an argument of the relational concept *cause to give food*.

### If Given Types of Categories Do Not Fulfill the Categorization Function (Thematic Categories Specifically), Do They Exist?

One of the most important functions categories have is that they allow transfer of knowledge which is beyond the available perceptual information (Komatsu, 1992; Hampton, 2001). According to *RoleMap*, this is done by mechanisms for creation of mappings between similar items and transfer through anticipations based on those mappings. As pointed in the beginning, there are various types of categories and what is common between them is that all members of a given category share some similarity. The members’ similarity might be between their features (as in the case of feature-based categories), the members might have similar role in a structure (as in the case of the role-governed categories), or they might share similar function for achieving a certain goal (as goal-based categories do).

Some researches argue that there is a specific type of relational categories, called thematic categories (Lin and Murphy, 2001; Estes et al., 2011). They differ from role-governed ones which members play the same role across different events. On the contrary, thematic categories group things together playing different roles in the same relation, even if they are from different situations. As such, thematic category members do not share the same function (or property). On the contrary, usually the members fit each other’s functions – as in the case of *dog* and *leash*. This makes them strongly connected to co-occurrence effects and to the situations in which they appear and interact (Gentner and Kurtz, 2005; Goldwater and Markman, 2011).

However, the thematic categories are not appropriate for the function, mentioned above. If, for example, a *pig* and a *grandmother* are both members of a category such as *at the village*, one should expect that such a category should produce a mapping between the *pig* and the *grandmother* and a lot of knowledge for the *grandmother* should be available for transfer to the *pig*. Note, *RoleMap* is a highly context sensitive model. In a specific context, mapping between *pig* and *grandmother* can occur, but this would be either if their commonality is on a very abstract level (i.e., both are *living things* in which case information about livingness would be transferred), or if the two play similar roles in common relational structures in which case, again, specific information would be transferred.

To be clear, *RoleMap* stresses on the existence of schema-governed categories. The model can abstract a category such as *to be in a village* for example. The members of this category would be different situations of being at a village. The prototype of this category may consist of *pigs*, *grandmothers*, etc., interrelated with relations. However, they would be **parts of the schema** and **not its members**. Technically speaking, in the notation of the *RoleMap*’s knowledge representation, in this case the links from the *pig*, *grandmother*, etc., to the schema-governed concept should be of *part_of* type (not of *is_a* type), while the category exemplars connect to the corresponding category via an *is_a* relation (as would be the case of *wolf* and *snake* both being/*is_a predator*).

However, there are empirical evidences supporting the claim that thematic categories exist in the sense that entities that share nothing in common, unless their co-occurrence, could be members of (and not parts of) categories that represent only their co-occurrence. Experimental designs, usually used in studies which support such claims, include three objects – a target and two alternative options, and people should either choose which of the two options is more similar to the target one, or give a common name for the target and one of the options (Lin and Murphy, 2001; Jones and Love, 2007; Goldwater and Markman, 2011).

According to *RoleMap*, similarity could be treated as a function of both associative closeness and structural alignment. Thus, the behavior interpreted as a result of the existence of thematic based categories could also emerge from associative closeness.

#### Simulating “Thematic Categories” Without Thematic Categories

We chose one of the experiments of Goldwater et al. (2016), (Experiment 2) to test whether *RoleMap* may simulate their data without explicitly postulating thematic categories. The researchers created 3 types of videos containing novel objects (situations of *chasing*, *pushing* and *lifting* each with three versions, totaling in 9 videos). All videos presented three objects, two of which were always interacting with each other (one of them *chased*, *pushed* or *lifted* the other), while the third one was moving impartially to the other two. All objects from the different videos that share the same role in one and the same relation (for example, all *chasers*), are members of a role-governed category. In turn, all objects from one and the same video are parts of one and the same situation. What was interesting was whether people group together different objects from different situations who participate in the same relation, but in different roles (for example, a *chaser* from one video and a *chasee* from another). If they do, one may think of these groupings as thematic categories.

After presenting the videos, the researchers gave to their participants a classic triad choice task in which they had to answer which one of the two given objects goes best with the target one to form a category. The target could have appeared with either: (1) an object of the same role and a bystander; (2) an object from the same theme and a bystander; or (3) an object of the same role and another one from the same theme. The results from this setting showed that people group either by role versus a bystander (56%); or by theme versus a bystander (58%). Finally, when the participants had to choose between an object that was in the same role or an object from the same theme, the role-governed option was preferred (57%) (Goldwater et al., 2016)^7^.

We modeled that experimental setting in the following way. First, we encoded semantic knowledge about three schema-governed concepts – *chasing*, *pushing* or *lifting* each of which had as parts its corresponding role-governed concepts and the relations between them. For example, the schema-governed concept *chasing* had as parts the relational concept *chases*, the role-governed concepts *chaser* and *chasee*, and finally, a *bystander*. The structured descriptions of the nine videos, presented to the participants were also encoded as base knowledge. Each video was represented through an episodic agent (instance of the respective schema-governed concept) and had as parts the objects from the video and the relations between them. That way, *RoleMap* had the knowledge that there are three instances of the role concept *chaser*, three instances of the role concept *chasee*, etc.

To test the model’s preference about which option corresponds best to the presented target one, we designed 18 test triads following the criteria used in the reported experiment and sequentially presented them to the model. An example role triad would be *chaser_1* (from the first video), *chaser_2* (from the second video), *bystander_1* (also from the first video). The target from each triad was set as target agent (which automatically raised the agent’s activation to 1.00) and the two options from the same triad were manually made active (also raising their activation to 1.00). In addition, we artificially attached correspondence hypotheses between each target and the two options from its triad. Depending on the triad, there were three types of hypothesis – (1) same role hypothesis – for example, two *chasers* from different situations; (2) same theme hypothesis – for example, a *chaser* and a *chasee* from different situations; and (3) participating in one and the same episode hypothesis – for example, a *chaser* and a *bystander* from one and the same situation. The first hypothesis received justification by the role concept the items were instances of – in that case, the role concept *chaser*. The second hypothesis was justified by the general schema-governed concept (*chasing*) which had as parts the two role-governed concepts (*chaser* and *chasee*). The last type of hypothesis was supported by the episodic agent representing the concrete situation the two items were part of.

We again repeated the simulation multiple times, randomly varying the test order and the added noise to the weight of all links [N(0, 0.25)]. For each test triad, we left the model do its usual processing for a predefined time of 13 cycles. At the end, we calculated the activation level of the three hypotheses. The most active hypothesis was interpreted as the model’s choice for the better correspondence between the target and the two available options. We obtained the similar pattern of results as in the psychological experiment. When the model was forced to choose between a role-governed or a bystander option, the level of activation of the same role hypothesis was higher in 77%s of the cases. When it was tested with a same theme condition (meaning that it had to choose between option representing an element from the same theme or a bystander), the theme hypothesis activation was higher in 61%s of the cases. Finally, when *RoleMap* had to choose between a same role option and a same theme option, the model preferred the same role in 71%s of the cases.

Obtaining similar pattern of results as the results from the psychological experiment without the explicit postulation of thematic categories shows that the behavior, which can be easily misinterpreted as led by theme-based categories, might be due to other pressures – such as the associative closeness for example.

### Demystifying the Inverse Base Rate Effect – The Work of the Constraint Satisfaction Network

We would like to emphasize that the *RoleMap*’s abilities are not limited to working with relational categories. The model lies on the assumption that a huge number (and maybe all) of the categories are defined by structures of relations. However, the way its mechanisms are supposed to work allows every kind of distributed representation of something to be either categorized as something known or into a newly created category.

Because of this, the model can adequately address a wide variety of classical psychological phenomena and we chose the *inverse base rate effect* (Gluck and Bower, 1988) to demonstrate that. People have proven to be extremely sensitive and reckon on the *base-rates* of the categories they are presented with. Given that all other conditions are held equal, faced with the task to classify properties supporting two categories, people usually choose the more frequently observed one. This is clearly seen in cases when the participants should learn two categories each consisting of two features – *category A* (with features *C* and *M*) and *category B* (with the same feature *C* and a new one *N*); and the members of the *category A* are presented three times more often than those of *category B*. During the test phase, if the participants are faced with the common feature *C* alone, they prefer to categorize into the more frequent category A (which is a manifestation of the classical base-rate effect). Regardless of this preference, a phenomenon known as the *inverse base-rate effect* shows something else. If the two unique features *M* and *N* are presented together, people prefer to categorize them as belonging to the rarer *category B*. Not surprisingly, this effect still has not met a satisfactory theoretical explanation (Markman, 1989; Johansen et al., 2007; O’Bryan et al., 2017; Don and Livesey, 2017). On the contrary, its counterintuitive appearance has spread to occupying the categorization modeling domain as well (Kruschke, 1996; Juslin et al., 2001). As implemented in the *ADIT* model, Kruschke’s explanation of the effect can be reduced to asymmetrical representation of the learned categories, due to a rapid attentional shift during their learning (Kruschke, 1996). On the opposite, the *ELMO* model (Juslin et al., 2001) emphasizes that the effect is due to decision-making, i.e., people first probe the better learned rule (about the more frequent category) and/or the most similar one (depending on the test case) and if the probe does not match, they just choose the other category. Unfortunately, both explanations have been criticized for being far from flawless (Winman et al., 2003; Johansen et al., 2007; O’Bryan et al., 2017).

We see as unnecessary the pulling of additional representational assumptions, nor decision-making ones, supporting the theoretical arguments behind the inverse base rate effect. We see the effect as due to two pressures – the pressure to map similar things and the pressure to map one thing to only one other thing (the 1:1 pressure). The effect results of the relaxation of a constraint satisfaction network of those supporting and inhibiting pressure links.

For example, if there is a recognized *tail* on the input, various *tails* from the long-term memory will become active. Some of them will create and support the anticipation that there is a *cat* on the input and the target *tail* is part of it. Others will create and support the anticipation that there is a *dog* on the input, etc. Everything else being equal, the activation of each anticipation is mainly a function of the number of mappings that support it. The more exemplars of a given category are extracted, the higher the support for the respective anticipation would be. Thus, naturally, the *base-rate effect* would occur.

**FIGURE 7:**
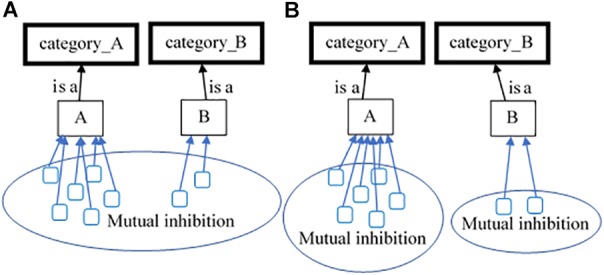
Demonstration of the base-rate **(A)** and the inverse base-rate effects **(B)** as naturally produced by the competing mechanisms in *RoleMap*. When a common property for *Category A* and *Category B* is given, all mappings inhibit each other **(A)** and *Category A* “wins”, because there are more mappings supporting it. However, when two unique properties are given, the inhibition is within each of the groups of mappings and not between the groups, thus, *Category B* “wins,” because the mappings that support it stronger (see text for details).

However, all contradictory mappings inhibit each other, because of the one-to-one correspondence, which puts pressure not to recognize one element as two different things. The pressure was not modeled for the *RoleMap*’s work specifically. On the contrary, it is assumed to be a basic principle of the cognitive system in general. Thus, a pressure, contrary to the one allowing the base rate effect, emerges – the more mappings a certain anticipation support, the weaker each of them is. This will not influence the example above – all mappings between the target’s *tail* and the *tails* from the memory will inhibit each other and they all will become proportionally weaker. This means that all anticipations will receive proportionally weaker support. However, if there are two elements on the input (for example, *meows* and *trunk*) and each of them will form a separate set of mappings, each set supporting only one anticipation (*cat* and *elephant*, respectively), the two distinct sets will not inhibit each other directly. The mappings supporting the *cat* anticipation will inhibit the mappings from the same set, without inhibiting the mappings supporting the *elephant* anticipation. Vice versa, the mappings supporting the *elephant* anticipation will inhibit each other (without inhibiting the mappings from the other set) (Figure 7). That will result in more, but weaker in support, mappings for the *cat* anticipation, and less, but stronger, for the *elephant*. In that case, the smaller set may win.

In summary, when presented with a feature that is shared by many categories, the most frequent category will have a categorization advantage. However, when there are two features each of which is representative for a different category, the frequency becomes a disadvantage. The one-to-one correspondence pressure makes it so more exemplars mean less power for each of the mappings supporting the respective anticipation^8^.

#### Simulating the Base Rate and the Inverse Base Rate Effects

To test this prediction, we initialized a knowledge base, consisting of two concepts – *A* and *B*. The concept *A* had 9 instances, all of them consisting of two features: *C* and *M*. The concept *B* had 3 instances, also consisting of two features: *C* and *N*. Each of the concepts’ instances was encoded as an episodic agent with two other agents as its parts, standing for the two features. On each run of the simulation, the model was tested with several patterns, sequentially presented in random order as targets. There were seven types of test patterns – (1) feature *C* only; (2) feature *M*; (3) feature *N*; (4) features *C* and *M*; (5) features *C* and *N*; (6) features *M* and *N*; (7) features *C*, *M* and *N*. Each target was presented and *RoleMap* classified it either as member of category *A* or *B*. The simulation was repeated 100 times with slightly different variants of the knowledge base – we added random noise to all links between the agents and to the agent’s initial activation [N(0, 0.25); N(0, 0.05), respectively].

The statistical results indicated that *RoleMap* behaved closely to human participants. When tested on the perfect predictors (feature *M* only and feature *N* only), the model classified them into the right category in 100%s of the cases, because it had no reason to anticipate the contradicting category. When *RoleMap* had to classify *C* and *M*, it chose category *A* in 100%s (when presented with *C* and *N*, it chose category *B* in 92.7%s of the cases). When tested only on the common feature *C*, *RoleMap* stuck to the classical base rate effect, classifying the feature *C* as belong to the high frequency category *A* in 85.3%s of the cases. Importantly, when presented with the ambiguous information (features *M* and *N* together), the model classified the features as belonging to the low frequency category *B* in 58.4 %s of the cases, which is a clear demonstration of the inverse base rate effect. When presented with all three features (*C*, *M* and *N*), the model anticipated that they belong to the category *A* in 70.3%s of the cases.

In summary, the simulation demonstrates that the inverse base rate effect may emerge as a side effect of the work of the constraint satisfaction network. That is, *RoleMap* does not need additional mechanism to account for all conditions associated with the base rate and the inverse base rate phenomena.

## Conclusion

We assume that the main function of the human conceptual system is to provide us with information that is important but could be unavailable in the moment we deal with it. To work well, such a system should be sensitive to various types of similarity, including structural ones. It should be as general as possible, meaning that keeping the same basic principles, it should be sensitive to different types of similarities, thus reflecting seemingly different types of concepts. Last, but not least, it should be context sensitive.

The simple stimulus-response association organized memory allows to react in similar way to similar stimuli. However, the environment is much more complex and evolutionary, probably more complex mechanisms of grouping various stimuli by similarity had emerged. The feature-based similarity seems more superficial and easier to be captured by direct perceptual system. However, the structural similarity is much more useful in certain situations.

There is no obvious reason to separate the mechanisms responsive for the generalization of different objects from those responsive for the combination of different events and situations into schema-governed concepts. They both serve one and the same main function – to transfer knowledge from memory onto the current situation.

However, even though the extension of the types of similarity has a lot of advantages, it may result in losing effectivity. Something that constraints the number of possible paths for categorizing by similarity, is necessary. Context dependency is a possible solution of this trade-off between flexibility and effectivity.

The cognitive architecture *DUAL* has been built on similar underlying assumptions, mentioned above, about the human cognition in general. Thus, it was natural to use it for the basis of the concrete implementation of the *RoleMap* model of categorization and category learning. The *RoleMap* model finds semantic and structural similarities and combines them both to decide whether to categorize a given input as something already known or to create a new concept. In a way, that seems similar to the work of *SEQL* (Kuehne et al., 2000). Where *RoleMap* differs is mainly in its context-sensitivity, which *SEQL* lacks. Some of the mechanisms of the model may seem unnecessary complex for achieving this aim. However, these mechanisms on their own are not designed specifically for categorization. Instead, most of them are thought as fundamental for many cognitive abilities. This gives advantages to *RoleMap* before all other models designed for working in special regime of categorization and category learning only (Love et al., 2004; Kemp et al., 2010). Instead, *RoleMap* is integrated with other models under the hat of the same architecture (Petkov and Shahbazyan, 2007; Petkov, 2013; Petrov, 2013), allowing the categorization and category learning to work together with all other cognitive processes.

A set of simulations was presented. The first few simulations simply tested whether the concrete implementations of the various pressures assumed work in the way we expected them to do. After that, we focused on an empirical challenge to the theoretical assumptions of the model. We demonstrated how *RoleMap* can account for findings serving as empirical arguments for the existence of categories that combine members without semantic nor structural similarity between them (Simulating “Thematic Categories” Without Thematic Categories). Finally, we highlighted the interplay between the modeled pressures (Simulating the Base Rate and the Inverse Base Rate Effects). This simulation served as an illustration of this interplay, but it also demonstrated how one puzzling till now empirical effect (the inverse base-rate effect) could be naturally explained in the terms of interaction of various mechanisms, none of them designed explicitly for modeling this effect.

In fact, the boundary conditions of the inverse base-rate effect and its potential explanations continue to be of interest (Don and Livesey, 2017). Aiming to further investigate the effect, Don and Livesey carefully manipulated the novelty of the transfer trials, the global frequency of each category and the frequencies of the different cues. An important next step for *RoleMap*’s development is to be tested against the new conditions.

The *RoleMap* model is an infant in its development. As every other model, it has a lot of drawbacks. For example, *RoleMap* lacks mechanisms for slow learning. The weights of the links are set as being constant (except for the random noise added to them in some simulations for obtaining statistical data) and fixed to predefined parameters. Further equipment of the model with mechanisms for slow statistical learning (respectively, forgetting, which Corral and Jones (2014) note as highly important), for error-driven learning (for the need of such see Love et al., 2004), as well as for capturing of some bottom up pressures will be of large advantage for it.

Combining slow weight-based learning with the abilities of the model to account for one-shot structure-based learning, could allow various additional empirical phenomena to be accounted for. For example, (Kurtz et al., 2013) (also Doumas and Hummel, 2013) showed that explicit comparison is very important for the learning of relational categories. It would be interesting to explore the role of retrieval with more complex simulations with *RoleMap*. Retrieval is associatively driven in the model, thus probably it will be more difficult for it to create the most adequate mappings when the necessary elements for it should be retrieved, in comparison with a situation of simultaneously presented structurally similar items. The play between the associative based fast retrieval and the slower structural alignment processes may account also to some of the Goldwater et al. (2018) findings.

Probably the largest drawback of the model is that its initial representations should be hand-coded. This is an issue, especially when the user is asked to give a name for the newly created concepts. For example, for the purpose of the proof-of-concept simulations we may call *animal care* a set of 4 or 5 agents. But in fact, humans probably represent this concept in a much richer way than a set of few simple relations. In other words, the biggest problem is not so much in the hand-coding itself, but in the attempt to use a simple set of relations with the names used by people to point for much richer conceptual representations. When *RoleMap* works with non-mnemonic names and abstracts a relational category from let’s say *relation_12* (X1, Y2) and *relation_12* (T1, Z3), for example, the pure mechanisms of the model can be highlighted better, without thinking about the hand-coding issues, but this would make the model difficult for describing. That is why our main purpose was to clearly define *RoleMap*’s mechanisms together with smaller in scalability simulations, which better allow the model’s description. As tested only with simple examples, the model’s scalability is still questioned and should be tested further on. Nevertheless, in principle we see no reason for the model not to work with large representations. Yet, to do this the model needs additional mechanisms for automatic encoding of representations. A possible way to approach that is to incorporate external ontologies into the long-term semantic memory of *RoleMap*.

The model lies on the serious assumption that the perceptual system is alone able to recognize and represent relations. Even though this assumption is still disputable, a lot of empirical data support the assumption that a lot of top-down pressures influence even the very low-level vision and also object-background segmentation. The same assumption is made by the *Probabilistic model of theory formation*, proposed by Kemp et al. (2010). Yet, the *Probabilistic model of theory formation* has the important shortcoming that its processing is highly cognitively implausible, as the authors note themselves (Kemp et al., 2010).

In fact, *RoleMap* has no mechanisms for learning of relations just through observing the environment without previous relational knowledge. Some initial set of relations are always hand-coded prior the model’s work. On the basis on the mappings of those relations the model can learn more abstract schema-governed categories or more concrete relations and role-governed categories. Some initial success in addressing the problem of learning structured representations from unstructured feature vector inputs has been reported by the researchers developing the *DORA* model (Doumas and Martin, 2018). Potentially, *RoleMap* can adopt similar approach to deal with this problem.

Finally, a large set of types of categories is still unaddressed by the model. Eventually, *RoleMap* can account for the theory-based and goal-driven categories (Barsalou, 1983; Goldstone, 1994), also probably for the probabilistic ones (Jung and Hummel, 2015). Goldwater et al. (2016) explores the increasing role of explicit comparison when the relational categories are based on higher order relations. *RoleMap* relies on the higher importance of the higher-order relations but also is based on associative based retrieval. Thus, it potentially can account for all these findings, however, specific simulations for these should be designed. In summary, despite of the lots of drawbacks that *RoleMap* still has, it contributes a lot for our understanding how the human categorization system works – it supports the idea that most of the concepts are not represented just by lists of properties, prototypes, or examples, but are systems of structured knowledge. It advocates the understanding that common underlying mechanisms for various cognitive processes should be searched. It emphasizes the role of the context on how people categorize; and finally, it accounts for the fast learning with only few examples.

## Author Contributions

Both authors have made equal substantial, direct and intellectual contribution to the work, and approved it for publication.

## Conflict of Interest Statement

The authors declare that the research was conducted in the absence of any commercial or financial relationships that could be construed as a potential conflict of interest.

## Footnotes

1 However, in addition to the perceptual characteristics reliance, the similarity could be context dependent (Goldstone, 1994); also, it could be based on cultural norms (Hampton, 2001; Kloos and Sloutsky, 2004), etc.

2 Most of the *RoleMap*’s mechanisms are inherited from the *DUAL* architecture. However, for simplicity, we will describe only the mechanisms implemented in *RoleMap*, without pointing to their history of development.

3 The INPUT and GOAL nodes, inherited from *DUAL*, allow modeling separately the environment and the goals. *RoleMap* can potentially use the GOAL node for simulation of the acquiring and the usage of various goal-driven (Barsalou, 1983) and nominal (Kloos and Sloutsky, 2004) categories. However, none of the simulations, described in this article, uses it.

4 See the implementation of the model on Python 3.5. at https://github.com/nbuDUAL/DUAL.

5 In general, *RoleMap* deals with relatively simple structures, which we call with mnemonic names. We don’t assume, however, that the rich human knowledge about *milking cows* could be captured by so simple structures. Thus, one cannot expect that the model accounts to all associations and additional knowledge that *milking a cow* schema may induce in people.

6 *RoleMap* will work the same way with names like *agent001*, *agent002*, etc. Also, the model can set formal names on its own, without any human intervention. Nevertheless, we used the mnemonic names for better description. This is better for reading the text but may cause other issues. For example, some people may disagree that the abstraction of feeding chickens and milking cows is proper to be named *caring for animals*.

7 See the original paper for more detailed statistical results.

8 Note, however, that exactly this property ensures a better chance something to be categorized as something known, if the system already has a lot of exemplars of the respective category. In this case the competition is not between two anticipations, but between single anticipation and the mappings bellow it in the part-of hierarchy.
