# Supplementary material for: Relation-Based Categorization and Category Learning as a Result From Structural Alignment. The RoleMap Model
Source: Front Psychol. 2019 Mar 20;10:563. doi: 10.3389/fpsyg.2019.00563 (PMC6435783; doi:10.3389/fpsyg.2019.00563)
Supplement: Supplementary file 1 [file Table_1.docx]

Supplementary Material

Relation-based categorization and category learning as a result from structural alignment. The *RoleMap* model

Georgi Petkov*, Yolina Petrova*

*Correspondence:

Georgi Petkov - [gpetkov@cogs.nbu.bg](mailto:gpetkov@cogs.nbu.bg); Yolina Petrova - [yolina.petrovaa@gmail.com](mailto:yolina.petrovaa@gmail.com);

# Supplementary mechanistic description of the *RoleMap* model for categorization

The cognitive architecture *DUAL* and the model *RoleMap* specifically are built up of distinct agents each of which combines symbolic and connectionist aspects. All agent types in the *RoleMap* model are under the head of the main agent class called DUAL agent. The DUAL agent class itself is subdivided into semantic agent and episodic agent which in turn have their own subtypes (see Table 1 for all types of agents together with the slots of information each of them holds):

| **Table 1 \| Types of agents and the slots of information each of them holds** | | | | | |  |  |
| --- | --- | --- | --- | --- | --- | --- | --- |
| **Agent type** | **Inherits from** | | **Slot information** | | **Intended purpose** | |  |
| *DUAL agent* |  | name, comment, activation level, list of incoming links, list of outgoing links, list of the parts (agents pointing with part_of links to the agent at hand), list of wholes (agents to which the agent at hand points to with part_of links), list of relations (agents of which the agent at hand is an argument); | | main holder of all agents | | | |
| *Semantic agent* | *DUAL agent* | list of instances, list of superclasses; | | represents all types of concepts (i.e. all types in the type-token differentiation); as such it is the holder of the following sub-types of agents: *Concept agent*, *Relation concept agent*,  *Mapping agent*; | | | |
| *Episodic agent* | *DUAL agent* | list of is_a agents, list of mappings (correspondences); | | represents all types of instances (i.e. all tokens in the type-token differentiation); as such it is the holder of the following sub-types of agents: *Instance agent*, *Relation instance agent*,  *Anticipation agent*; | | | |
| *Concept agent* | *Semantic agent* | list of instances,  list of subclasses; | | represents all types of concepts, without the relations; currently, all *Concept agents* are hand-coded (except for the mapping agents transformed into *Concept agents*, which *RoleMap* does itself); | | | |
| *Relation concept agent* | *Semantic agent* | list of instances, list of subclasses, list of arguments, arity; | | represents all long-term relations; currently, all *Relation concept agents* are hand-coded (except for the *Mapping agents* transformed into *Relation concept agents*, which *RoleMap* does itself); | | | |
| *Mapping agent* | *Semantic agent* | target agent, base agent, list of superclasses (contains the common for the target and base agent superclass, if there is such), list of justifications, list of competing mappings; | | represents correspondences between two episodic agents – a target and a base one; all *Mapping agents* are created by *RoleMap* itself and are treated as proto-concepts as some of them could be transformed into long-term *Concept* and *Relation concept agents*; | | | |
| *Instance agent* | *Episodic agent* |  | | represents all types of instances, without the relations; currently, all *Instance agents* are hand-coded (except for the *Anticipation agents* transformed into *Instance agents* and the re-categorization of base knowledge, which *RoleMap* does itself); | | | |
| *Relation instance agent* | *Episodic agent* | list of arguments, arity; | | represents all long-term instance relations; currently, all *Relation instance agents* are hand-coded; | | | |
| *Anticipation agent* | *Episodic agent* | list of justifications, list of competing anticipations; | | represents the category belonging anticipations that *RoleMap* creates itself; the *Anticipation agents* are treated as proto-instances as some of them could be transformed into long-term *Instance agents*; | | | |

In fact, *RoleMap* operates only over the 6 sub-types semantic and episodic agents (those are concept agents, relation concept agents, mapping agents, instance agents, relation instance agents and anticipation agents). The main agent types – DUAL agents, semantic and episodic agents – are holder classes from which the rest of the agent types inherit slot information. In other words, each agent sub-type contains all the slot information that its upper class contains (i.e. all agents have a unique name – an attributed inherited from DUAL agent; just as all semantic sub-type agents have a superclasses slot, inherited from the semantic agent object class).

The simulations done in *RoleMap* work as follows:

1. **Hand-coding of semantic memory**

First, a set of concept and relation concept agents, forming a kind of small ontology, is manually encoded. In this phase, the researchers could create several types of links between the hand-coded semantic agents, thus filling up the links’ corresponding slot information. Table 2 presents a list of those link types and their default.

| **Table 2 \| Permanent links between the long-term agents construing the semantic and episodic memory of the *RoleMap* model** | | |  |
| --- | --- | --- | --- |
| **Type of link** | **Intended purpose** | **Link weight** | |
| *is_a* | from a more concrete to a direct more abstract concept; as well as from an instance to a concept it instantiates; | *0.5* | |
| *reversed is_a* | from more abstract to a direct more concrete concept; as well as from a concept to its instance; | *0.3* | |
| *part_of* | from a part to its whole; | *0.5* | |
| *has_parts* | from a whole to its part; | *0.3* | |
| *argument* | from a relation to its argument; | *2.0* | |
| *argument_of* | from an argument to the relation that it is an argument of; | *2.0* | |

For the relation concept agents, the arity slot is filled with a whole number depicting the number of the relations’ arguments.

When an ontology is manually encoded, its content follows a principle of consensus among the research team – aiming at representing the natural intuition about the semantic richness that people have.

1. **Hand-coding of episodic memory**

Following the first step, next hand-coded are set of instance agents and relation instance agents, representing a kind of small episodic knowledge/small number of episodic situations. Again, only the links included in Table 2 are filled. Accordingly, each arity slot of each relation instance agent is filled with a whole number depicting the number of the relation’s arguments.

Importantly, at first there are no single agents representing whole situations, meaning that the distinct episodes are not explicitly separated from each other. This reflects our assumption that the interconnections among the agents themselves implicitly represent the boundaries between the different episodes. During the model’s work, however, *RoleMap* automatically creates instance agents explicitly representing whole situations (this functionality is described in more detail in section 5.4 below).

1. **Hand-coding of the target episode**

The encoding of the target episode follows the principle of the base memories’ encoding. Yet, there are two additional functionalities regarding the target agents: First, they are entered in a global variable which allows the creations of mappings only between a target and a base agent. Second, the target agents entered in a global variable INPUT, which makes their activation level equal to 1.00 during the whole run of the simulation (i.e. the target agents are highly activated at the very beginning of the simulation, becoming a constant supply for the activation to the whole system).

1. **Adding noise to the weights of some of the links and to the initial activation of some of the agents**

Before running the simulation, some random noise is added to the weights of the connections between the agents and to the agents’ initial activation. This is done in order to achieve statistical results and to simulate randomly slightly different states of the model. During the simulations reported in the manuscript, we added a noise from N(0, 0.25) to *all* links created during the previous steps of the hand-coding of the knowledge. Random noise from N(0, 0.05) was also added to the initial activation levels of all already encoded agents (except for the target agents).

1. **Run of the program**

The whole cognitive architecture *DUAL* and the model *RoleMap* specifically work through repeating cycles. On each cycle *RoleMap* does several sub-cycles over the defined agents. The sub-cycles are: (1) calculating the net input for each agent; (2) calculating the new activation level of each agents; (3) removing the agents, whose activation level has dropped below the pre-defined threshold of 0.2 from the working memory (WM); (4) entering into the WM of the agents, whose activation just exceeded the pre-defined threshold of 0.2 for being into the WM; (5) handling of the agents which just entered into the WM (depending on their type) which includes the formation of requests for creation of new agents – as mappings and/or anticipations; (6) handling the requests – checking for duplicates, combining of justifications, inhibitions, and others; and finally (7) checking for winners among the temporary agents (mapping and/or anticipation agents) and eventually, transforming them into permanent agents (concept, relation concept, and/or instance agents).

**5.1. Spreading of activation**

***5.1.1.*** In each cycle over the model’s agents, each agent calculates its own net input (the sum of the activation level of its neighbors, multiplied by the weights of the links connecting them).

***5.1.2.*** In each cycle over the model’s agents, each agent calculates its new level of activation.

The activation function is a weighed sum of the old activation level and the net input of all its incoming links (depending on its type, each link has a specific weight – see Table 2 and Table 3) multiplied by a decay parameter:

**new_activation = decay_rate_parameter * (w * old_activation + (1 - w) * net_input)**

The parameters are fixed as following: *decay rate parameter*: 0.90; weight for the old activation level *w*: 0.75; respectively weight for the net input *(1 – w)*: 0.25.

Each agent stores its renewed level of activation in a specific slot. If it happens that the new activation of a certain agent just exceeded the threshold for entering in the WM (threshold parameter = 0.2), then this agent is added to a global variable containing the agents which just entered the WM.

***5.1.3.*** A cycle among the global variable containing all agents active enough to be in the WM during the previous cycle, checks whether in the cycle at hand there are agents, whose activation level has dropped below the pre-defined threshold (threshold parameter = 0.2). If there are such agents, they are included into a global variable containing all agents which should be removed from the WM.

**5.2. Handling the agents exiting and entering the WM**

***5.2.1.*** A cycle through the global variable containing the agents which should be removed from the WM removes those agents from the WM. In parallel, it takes care of all the necessary consequences such as removing its mappings from the WM, the requests for the creation of novel agents (if there are any), all temporary links connecting that agent to the rest of the active memory, etc.

Through a sub-cycle over the global variable containing the agents which just entered the WM, depending on the type of the agents, *RoleMap* does the following things:

***5.2.2.*** If an instance agent or relation instance agent enters the WM, the model is pressured to extract any semantic similarities between that agent and other instance or relation instance agents from the WM, i.e. looks for potential mappings/correspondences. To be a potential correspondence, certain conditions should be satisfied: (a) to be from the opposite kind of the target-base pair; (b) to share common semantic agent upward the is-a hierarchy; (c) all agents construing this path along the is-a hierarchy that connects the two agents should be in the WM.

***5.2.3.*** If a concept agent or relation concept agent enters the WM, the model checks whether this agent closes a WM path connecting two instance or relation instance agents. If it does, *RoleMap* forms a request for a novel mapping agent to be created.

***5.2.4.*** If a mapping agent enters the WM, RoleMap check whether its base and target agents are relation instance agents. If they are, through the structural correspondence pressure, the model forms requests for creation of new mapping agents between respective arguments of the two relations. In this case, the mapping agent, which represents the correspondence between the two relation instance agents, acts as a justification of the requested mappings.

In addition, the mapping agents entering the WM check for any competitors (i.e. other mappings that contain the same target agent). If there are such, *RoleMap* creates bidirectional inhibitory links between all competing mappings (see Table 3 for the default strengths of the links).

Finally, each mapping agent which enters the WM checks whether it can contribute to filling up any missing information in the target episode. Importantly, for *RoleMap*, the anticipations of interest are those that categorize the target as a situation. In other words, if a part of the distributed representation of something on the input is mapped to a part of an already categorized situation, an anticipation denoting the category of this situation particularly is created. More specifically, to do this the mapping agent goes through the *part_of* links of its base agent and checks whether its *part_of* neighbors have respective corresponding agents among the target’s *part_of* neighbors. In case there is a missing element in the target, *RoleMap* forms a request for a new *anticipation agent* which, after creation, would be supported/justified by the mapping agent – meaning that a justification link between the two would be established (again, see Table 3 for the default strengths of those links). If such anticipation agent already exists, the mapping does not request the creation of a new anticipation agent, rather a justification link between the two is formed.

| **Table 3 \| Temporary links construing the constraint satisfaction network** | | |
| --- | --- | --- |
| **Type of link** | **Depicted pressure** | **Link weight** |
| *instance to/from mapping* | bias to find correspondences | *0.5* |
| *concept to/from mapping* | justification for correspondence | *0.5* |
| *supporting link from mapping to mapping* | systematicity and bias to higher order relational thinking | *0.5* |
| *inhibitory link from mapping to mapping* | 1:1 correspondence | *-0.5* |
| *concept to/from anticipation* | bias to classify | *0.5* |
| *supporting link from mapping to anticipation* | Justification for classification | *0.5* |
| *inhibitory link from anticipation to anticipation* | 1:1 correspondence | *-0.5* |

***5.2.5.*** If an anticipation agent enters the WM, the agent looks for competitors (i.e. other anticipations supported by mappings containing the same target agent). If there are such, *RoleMap* creates bidirectional inhibitory links between them. In addition, the anticipation agent checks for mappings that might support it.

(Note, all agents work independently of each other. Thus, in general, some mapping agents should support some anticipation agents. However, there is no guaranty which agents will be created (and enter the WM) first – sometimes those are Mapping agents, sometimes anticipations. That is why, the need of adding justifications from mapping agents to anticipation agents should be checked both when a mapping agent enters the WM and when an anticipation agent does. This principle is valid for other operations as well. For example, such is the case with the search for an active path through the class hierarchy between two instances. Such path is searched for both when an instance or a relation instance agent enters the WM and when a concept or relation concept agent does (return to 5.2.2 and 5.2.3. for more details).

**5.3. Creation of new temporary agents (requests’ revision)**

Next, *RoleMap* performs a sub-cycle over all requests for creation of novel temporary agents (there are two types of temporary agents in *RoleMap* – mapping and anticipation agents). The handling of the requests starts with checking for duplicates among them. There are different procedures defining what is a duplicate depending on the type of the agent at hand. For a mapping duplicate is considered a mapping which shares the same target and base agents. If such a request is being handled, then the model does not create any new agents. Rather, the justifications (the superclasses and the inhibitions, if there are any) are transferred to the already existing one.

In case there is a mapping request representing a semantic similarity and the request is not a duplicate, the following happens. A mapping agent is being created. A mapping agent created in this way is linked to the two corresponding base and target agents. Respectively, the common concept of the two corresponding agents is set as a superclass of that mapping agent (such are the cases described in sections 5.2.2 and 5.2.3.). The other wat in which a mapping agent can be created is through finding a structural similarity (as described in section 5.2.4.). If a request for the creation of such mapping is being handled and that request is not a duplicate, the new mapping agent will again be linked to the two corresponding base and target agents. However, the superclass slot will remain empty. In case a new mapping request concerning the same base and target agents but representing a semantic similarity at a later point appears, the respective superclass link will be created, and the slot will be filled with that agent.

Anticipation duplicate is such an anticipation which offers the target agent to be categorized as part of one and the same category. If the request does not refer to an existing instance agent, an anticipation agent is created. If it is a duplicate, the model does not create a new agent. Rather it transfers its justifications to the already existing one.

**5.4.** **Creation of new permanent agents (transformation of mapping and anticipation agents)**

Finally, *RoleMap* performs a sub-cycle over all mapping and anticipation agents and checks whether the activation of any one of them exceeds a predefined threshold for transforming it into a permanent agent (default value of the threshold parameter = 10.0). There are three types of permanent agents that *RoleMap* creates by itself – those are concept, relation concept and instance agents.

If it happens that a mapping agent exceeds the threshold, *RoleMap* transforms that mapping agent into a concept agent (if the mapped agents are instance agents) or into relation concept agent (if the mapped agents are relation instance agents). The new agent inherits the superclass of that mapping (if there is such), while the mapped target and base agents are set as the first two instance agents of the newly created concept or relation concept agent. This denotes the category learning process. At the end of that process, destroyed are the mapping agent at hand, its competitors and all anticipation agents that the mapping agent was justifying (so the categorization tendency is stopped).

Importantly, the newly created permanent agent supports the transformation into permanent concepts and/or relation concepts of some of the other mappings. Those are the mappings of the other target agents, which are interconnected with relations with the target agent of the just transformed mapping into permanent agent. All these newly created concepts and/or relation concepts form a coalition of agents. This coalition is the distributed representation of a new entity, which binds the whole relational structure on the input. The model creates one additional concept agent, representing this entity – capturing the what is called schema-governed concept. This schema-governed category might be thought of as kind of a binding node for the lower level concepts of the part-of hierarchy. Thus, when a new schema-governed concept is created, it emerges both with its distributed and localist representation. In addition, also created are two new instance agents – one binding the interrelated target episode and another one binding the target’s corresponding base episode. Those two instance agents become the schema’s first exemplars (the first instances of the concept agent).

If it happens that an anticipation agent exceeds the predefined threshold, it is transformed into a new instance agent, denoting the categorization process. This new instance agent stands for a binding node for the elements on the lower level of the part-of hierarchy (the agents representing the target episode), whose mappings justified the anticipation. After this transformation, the model does some additional work to achieve consistency. The agents from its distributed representation adjust their *part_of* and *is_a* links consistently with the categorized situation. Respectively, their mappings are removed in order to stop their tendency of becoming permanent concept and/or relation concept agents.
